# A before and after cross-sectional analysis of a public health campaign to increase kidney health awareness in a Canadian province

**DOI:** 10.1186/s13104-015-1662-2

**Published:** 2015-11-20

**Authors:** Krista Ryz, Navdeep Tangri, Mauro Verrelli, Jan Schneider, Amie Lesyk, Amanda Eng, Brett Hiebert, Reid H. Whitlock, Manish M. Sood, Claudio Rigatto, Paul Komenda

**Affiliations:** Department of Medicine, Section of Nephrology, University of Manitoba, 66 Chancellors Cir, Winnipeg, MB R3T 2N2 Canada; University of Manitoba, Seven Oaks General Hospital, 2300 Mcphillips St, Winnipeg, MB R2V 3M3 Canada; Manitoba Renal Program, Winnipeg Regional Health Authority, 2300 Mcphillips St, Winnipeg, MB R2V 3M3 Canada; St. Boniface General Hospital, 409 Taché Ave, Winnipeg, MB R2H 2A6 Canada; Health Sciences Centre, 820 Sherbrook St, Winnipeg, MB R3A 1R9 Canada; Department of Community Health Sciences, University of Manitoba, 66 Chancellors Cir, Winnipeg, MB R3T 2N2 Canada; Department of Medicine, The Ottawa Hospital Research Institute, University of Ottawa, 725 Parkdale Ave, Ottawa, ON K1Y 4E9 Canada

**Keywords:** Kidney disease, Awareness public, Kidney failure, Health, Health promotion, Evaluation

## Abstract

**Background:**

Chronic kidney disease (CKD) has a major impact on patient health and health system resources. The prevalence of kidney disease is increasing, with Manitoba being one of the provinces in Canada with the highest per capita rate of CKD and end stage renal disease (Anonymous, Canadian organ replacement register annual report: treatment of end-stage organ failure in Canada, 2001–2010, 2011). In 2011, a public health campaign to promote kidney health, by increasing awareness of CKD and its risk factors, was created to target high-risk individuals such as First Nations and those with hypertension and diabetes in urban and rural/remote Manitoba. In this study, we aimed to determine the effectiveness of this public health campaign on increasing the awareness of CKD.

**Methods:**

Our public health campaign ran in March 2011, and employed a multifaceted approach with radio, television, internet, and print advertisements. Campaign awareness and understanding of the public health message were assessed with a telephone omnibus survey of randomly selected individuals with a Manitoba area code during February and April 2011. A before and after cross-sectional analysis was utilized to measure the effect of exposure to the campaign in telephone respondents.

**Results:**

1606 individuals participated in the survey (804 pre and 802 post). Overall awareness of the campaign messaging increased from 7 % pre campaign to 25 % in the post campaign period. Approximately two-thirds of respondents correctly identified a main theme message of the campaign. Awareness improved across most subgroups surveyed aside from those with lower education and income.

**Conclusions:**

Our study demonstrates the effective reach of our campaign and its relative effectiveness at raising awareness of chronic kidney disease and its risk factors.

## Background

Progressive chronic kidney disease (CKD) and kidney failure are a major worldwide public health problem. Risk factors for CKD are well described [2–6] and include diabetes, hypertension, and family history. Novel risk prediction algorithms for CKD progression suggest that when estimated glomerular filtration rate (eGFR), quantified proteinuria measures, age, sex and other common biochemistry tests are combined, kidney failure risk can be reliably determined [7]. Despite medical advances to detect and treat kidney disease, as well as predict its progression, many patients are still unaware of their CKD. Evaluation of over 100,000 participants in the National Kidney Foundation’s Kidney Early Evaluation Program revealed a CKD prevalence rate of over 25 %, but only 9 % of individuals were aware of this diagnosis [8]. Without individual patient awareness and participation it will be difficult to affect health outcomes associated with progressive CKD.

The province of Manitoba has among the highest kidney failure rates in Canada [1]. In 2010, the Manitoba Renal Program (MRP) designed a public health campaign with two major objectives: (1) increase knowledge of CKD risk factors, and (2) promote high-risk individuals to seek medical attention for an assessment of their kidney function. The campaign was run over the month of March for two consecutive years (2010 and 2011), and included radio, print, television, bus, and web based advertising. The two campaigns were very similar in terms of messaging and format. The impact of the campaign on CKD awareness in 2010, remained unknown. Evaluation of a public health campaign is important to determine health and behavior impact and economic effectiveness. In order to evaluate the success of our 2011 campaign, we conducted a cross sectional survey based study to examine awareness of CKD.

## Methods

### Study design

A before and after cross-sectional analysis was done of a random sample of the adult general population in this Canadian province to measure the effect of exposure to the campaign. Research was carried out in compliance with the Helsinki Declaration. As this was a quality assurance evaluation of a public health campaign, human ethics approval was not required by our research ethics board at the University of Manitoba.

### Population

Manitoba is a Canadian province with several unique demographic features. It is home to just over 1.2 million people, covering an area roughly twice the size of the United Kingdom [9]. In addition to a very low population density, Manitoba is also distinguished by having a large First Nations community. Based on 2006 census data, Manitoban First Nations people account for 15 % of the population, but unfortunately this group disproportionately represents 31 % of the individuals on hemodialysis [1, 10]. Twenty percent of the First Nations population live in either a rural or remote Manitoban community [9], thus it was important for our campaign to extend its reach broadly into those communities. Our campaign targeted high-risk populations for kidney disease including First Nations in urban and rural/remote areas during the month of March 2011. The omnibus survey included those age 18 years of age and older with a Manitoba area code.

### Intervention

A multi-faceted public health campaign was undertaken in urban and rural/remote Manitoba, Canada in March 2011. The March 2011 campaign was built on a similar advertising platform that had been executed in 2010. Formats used to reach high risk individuals included: (1) Radio—daily messaging in English, Ojibway, Cree, Tagalog, Punjabi, and Mandarin, (2) Transit—king sized advertisements on side of buses in the city of Winnipeg, (3) Television—daily advertisements on the local weather channel, (4) Print—First Nation newspapers; postcards delivered with prescriptions through major pharmacy providing prescriptions to over 20 remote northern First Nation’s communities, (5) Website—kidneyhealth.ca, the MRP’s home website updated to include campaign imagery and message. Specifically, the printed advertising message included “Kidney Disease—know the risks” and denoted such risk factors such as diabetes, high blood pressure, heart disease and family history of kidney disease.

### Evaluation

**Fig. 1:**
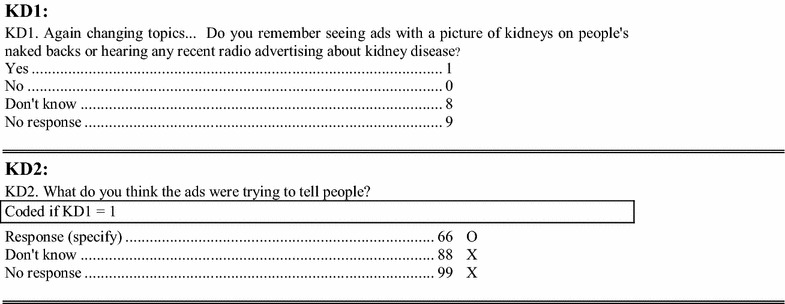
Manitoba Omnibus survey April 2011: research on kidney disease advertising

A pre and post campaign telephone omnibus survey of Manitobans age 18 years of age and older was performed in February and April 2011 respectively. Respondents were selected by random digit dialing and both mobile and land line phones were contacted. Each respondent was asked a screening question to determine awareness of the campaign (See Fig. 1 for initial survey questions) If aware, a follow-up question was asked to determine the individual’s understanding of the campaign message (Table 1). Basic demographic information was also collected on those that were aware of the campaign (sex, urban vs. rural, age category, level of education, and income quartile).

**Table 1:** Identification of key themes of campaign among aware respondents

Theme	February 2011 % (N = 56)	April 2011 % (N = 192)	*P* value
Raise awareness of kidney disease	16 (29)	57 (30)	0.87
Take care of your kidneys or health	16 (29)	37 (19)	0.14
Get checked for kidney disease	6 (11)	24 (13)	0.72
Support the kidney foundation	–	26 (14)	–
Donate organs	–	25 (13)	–
Diabetes-related message	–	6 (3)	–
Kidneys are important	1 (2)	4 (2)	1.00
Other	5 (9)	7 (4)	0.11
Don’t know/no response	17 (30)	35 (18)	0.05

Respondents could provide more than one response; totals may sum to more than 100 %

### Data analysis

Weighting was applied to data correcting for differences between the demographics of the sample and the 2006 census data on Manitoba’s population. We used descriptive statistics, a two-sample z-test to compare sample proportions (Table 1) and a Chi-squared test for categorical variables (Table 2) to examine the awareness of CKD and its risk factors pre and post campaign across some key demographic subgroups namely age, sex, urban vs. rural residence, income and education level.

**Table 2:** Subgroup analysis of factors influencing pre and post-intervention^a^

Variable	Pre-intervention (n = 804)		Post-intervention (n = 802)		Improved pre vs. post
	Aware	Not aware	Aware	Not aware	P value
Sex					
Male	24	321	80	256	<0.0001
Female	32	417	112	345	<0.0001
Location					
Winnipeg (urban)	44	430	133	336	<0.0001
Non-winnipeg (rural/remote)	12	309	59	265	<0.0001
Age group					
Age 18–39	19	242	74	159	<0.0001
Age 40–64	25	343	79	275	<0.0001
Age 65+	11	152	39	167	0.0007
Educational attainment					
<High school	7	88	11	70	0.1800
High school	11	285	67	219	<0.0001
University/college graduate	36	348	110	294	<0.0001
Household income					
Under 35 k	14	116	24	104	0.0700
35–75 k	10	230	72	178	<0.0001
Over 75 k	19	219	53	183	<0.0001

^a^Some respondents declined to give certain or all demographic information

## Results

### Campaign awareness

**Fig. 2:**
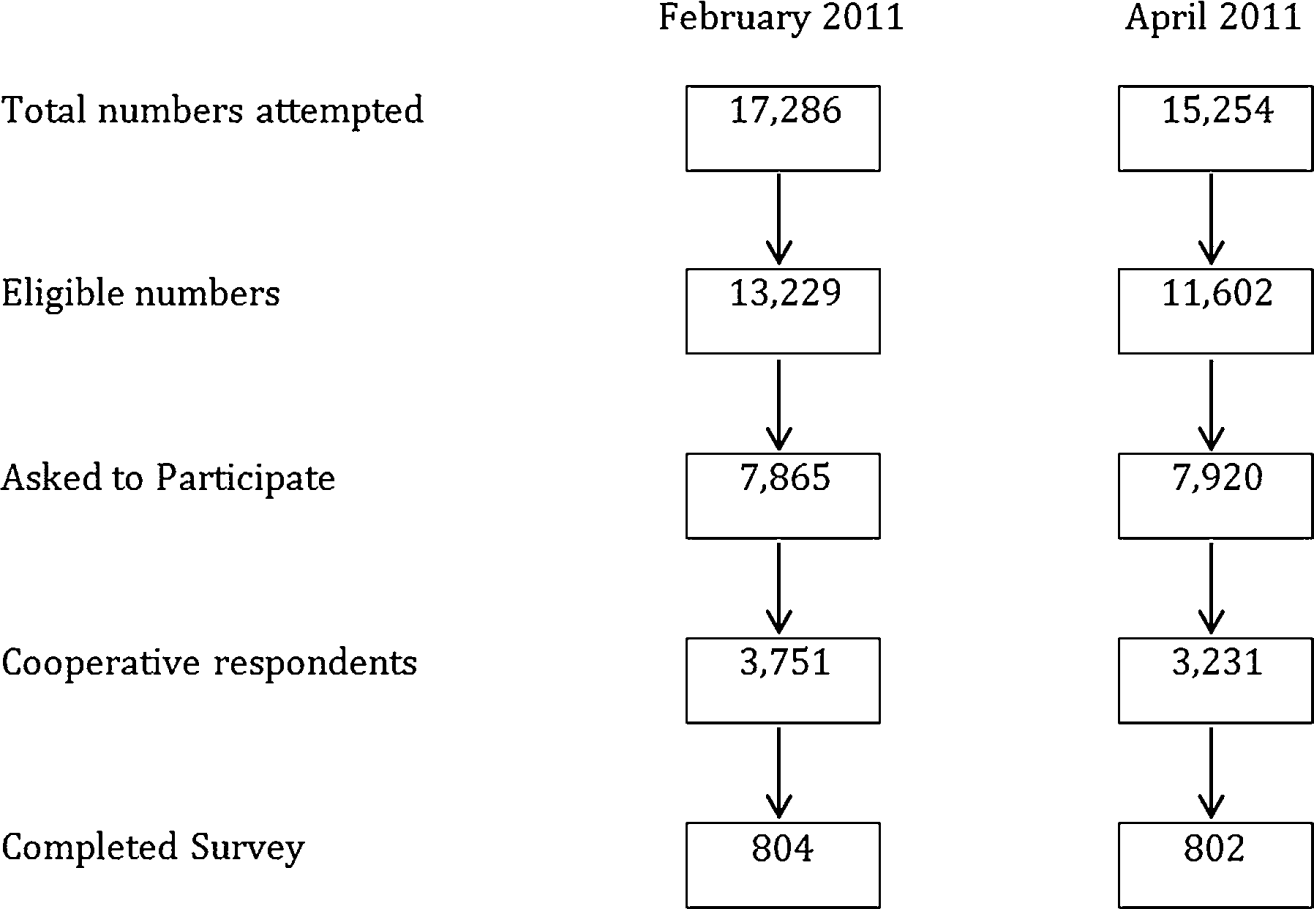
Cohort derivation

**Fig. 3:**
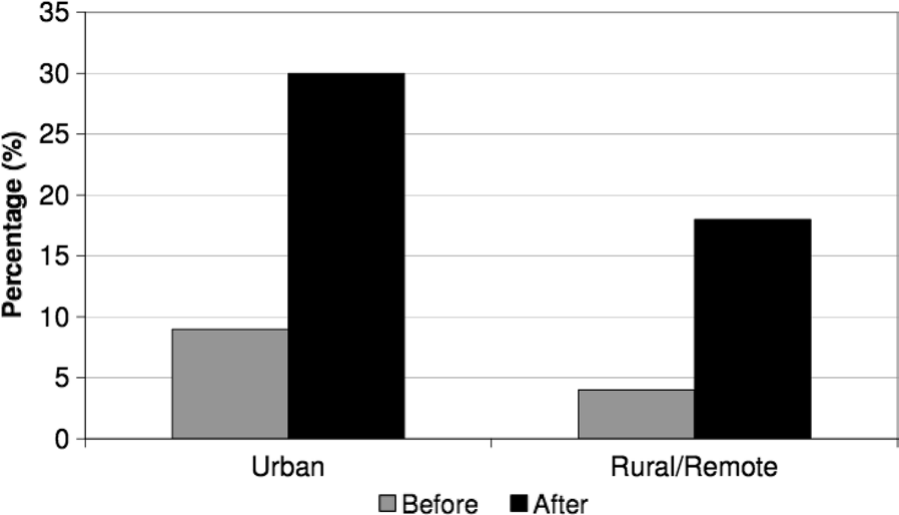
Awareness by region

**Fig. 4:**
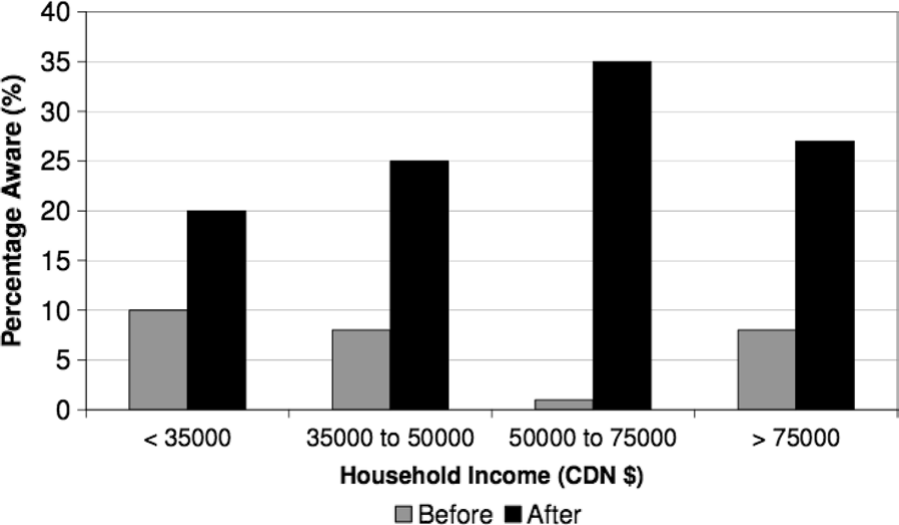
Awareness by income level

There were 17,286 numbers attempted to be dialed pre-campaign and 15,254 post- campaign. Out of the eligible contacts who were asked to participate, there were a total of 3751 pre-campaign and 3231 post-campaign contacts who cooperated. This led to a total of 804 qualified contacts pre-campaign, and 802 qualified candidates post-campaign that completed the survey (Figs. 1, 2). Overall, the percentage age of respondents that answered “yes” to the awareness question increased from 56 (7 %) to 192 (25 %) from pre to post campaign (p < 0.0001). The awareness of the campaign was increased in all groups studied including urban and rural/remote respondents (Fig. 3) and income levels (Fig. 4). Significant improvements in awareness were seen post campaign across virtually every subgroup except for those with less than a high school education and earning less than 35,000 dollars per year (Table 2).

### Message uptake

Respondents were asked to specifically identify themes of the campaign without prompting. These responses were categorized, and the main themes recalled included: (1) know the risk factors for chronic kidney disease (diabetes, hypertension, and family history), and (2) see your health care provider to have your kidney function assessed. In those that recalled the campaign, 39 participants were able to identify a key theme of the advertising campaign from the previous year, compared to 157 post campaign in 2011. However, there were no statistically significant differences in the key messages that were absorbed by individuals in the pre and post campaign survey (Table 1).

## Discussion

Progressive kidney disease and kidney failure is increasing at alarming rates [1, 11–13]. Despite our ability to successfully screen patients and predict progression, many patients are still unaware of their disease and risk [8]. We designed a multi- faceted public health campaign targeting high-risk populations, in an attempt to increase awareness of CKD risk factors, and promote patients to seek out medical care for screening. Evaluation of our results confirms awareness was successfully increased from pre to post testing between two and threefold. Although the identification of key message themes did not significantly change in the post campaign population, identification of a theme was found in almost two-thirds of participants. Awareness was improved in virtually all subgroups surveyed aside from those with less than a high school education, and earning less than 35,000 dollars per year; potentially the most vulnerable sector of the population for CKD.

Public health campaigns targeting individuals have been able to significantly impact the health behaviours of populations. Meta-analyses have shown the average effect size on behaviour change that can be expected from an individual campaign is roughly 5–9 % [14, 15]. However, the impact of individual campaigns ranges from dramatically influencing the intended audience to bearing no effect [15]. Thus, evaluation of any public health initiative is a key element to successful health promotion. Without this critical step the success (or lack thereof) of the initiative, cost-effectiveness, and planning for future strategies is impaired. This step has become routine in public health campaigns targeting HIV prevention, Hepatitis C awareness, nutrition interventions, and family planning campaigns amongst others [14, 16–19]. However, there has been a scarcity of literature on the evaluation of campaigns targeting kidney disease and kidney failure. One study was found that examined the effect of World Kidney Day (WKD) on the awareness of CKD in at risk individuals in a Korean cohort [20]. They were able to show that WKD campaign translated into an overall increase in awareness of CKD of 4.7 %, but overall awareness still remained low and thus indicates that further investment in kidney disease related public health campaigns is warranted.

The 17 % age point increase in population awareness of our campaign was much greater than the average effect size expected. Several factors may have contributed to this outcome. These may include the ability of the campaign to access its intended recipient, known as the campaign’s reach, and the frequency of message delivery to the intended audience. Due to large geographic distribution of the audience and the diversity of ethnic groups at high-risk for kidney disease, many facets of media were employed during our campaign including radio, television, internet, and print advertisements. In addition, multiple languages were used to increase reach. Evaluation has shown that campaigns with high diversity in mode of message delivery coined “big messy campaigns” tend to be more successful [15]. Thus, our multi-pronged approach may have contributed to an increased reach and frequency of the campaign. Also, duration of the campaign itself may have had a positive impact on increasing awareness as shorter, intense campaigns have been associated with increased reach and effect size [21].

Our multi-faceted province-wide public health campaign was effective at reaching our target audience; however, the identification of key themes was not significantly altered from February to April 2011. There are a variety of factors that may explain why theme identification did not parallel the increase in awareness seen. Firstly, it is important to note that theme identification was already at a high level based on pre campaign testing. This may indicate the success of previous years’ similar campaigns at targeting our audience. Factors that may have interfered with theme identification may include message complexity, language barriers, and literacy barriers. Efficacy of the various arms of our multi-faceted media campaign were not evaluated individually, therefore, it is difficult to know if the attempts to overcome literacy and language barriers (radio and television messaging in a variety of languages) were effective. Focus groups with members from the target audience could clarify understanding of the message and help tailor the campaign in future years. In addition, the populations with the highest level of awareness were young, urban, affluent respondents. This may reflect a population with a higher degree of education, literacy, and computer use. Methods of reaching our high-risk population that do not have these skills or computer access need to be explored.

### Limitations

Evaluation of our campaign revealed several important limitations. When comparing awareness and theme identification of subgroups among pre and post intervention survey participants some numbers were too small to draw any firm conclusions. Our evaluative process did not capture demographic data on ethnicity. Random digit dialing was used to select respondents and therefore, theoretically, should evenly represent the distribution of ethnic groups in the province. However, in reality there may be underrepresentation of some groups. The evaluation survey was done in English language, and thus, may have excluded high-risk individuals in which language was a barrier. By the nature of a telephone survey as an evaluative tool our data would have excluded individuals that do not have telephone access. Finally, it is possible that some of the increase in awareness may not translate into a change in behaviour. This is known as the knowledge gap and thus the true effect size on behaviour may be smaller than anticipated from the documented increase in awareness [14].

### Strengths

There are several important strengths of this study to highlight. First, this report is one of the only published accounts of an evaluation of a public health campaign to target awareness of CKD and its risk factors. Although there are a few published accounts of public health promotion activities in nephrology, these have often lacked the critical step of evaluating the impact of the efforts [22, 23]. In addition, we have shown that using a multi-faceted campaign we can successfully overcome geographic challenges of promoting health in a large area with low population density.

## Conclusions

A public health awareness campaign on CKD and its risk factors increased familiarity. In addition, identification of key themes remained at high levels. The campaign was more effective at reaching an urban, younger, and more affluent audience. Further research will need to be done to evaluate the most effective way to improve on theme identification, to assess the economic impact of the campaign, reach more vulnerable populations and to assess for effects of the campaign on individual’s behavior and kidney disease outcomes. An important step in public health change is evaluating the effectiveness of any strategy undertaken and the omnibus tool is one method of doing so.

## Abbreviations

CKD: chronic kidney disease
eGFR: estimated glomerular filtration rate
MRP: Manitoba Renal Program
WKD: World Kidney Day
